# Editorial: Identification, function and mechanisms of interferon induced genes associated with viruses

**DOI:** 10.3389/fimmu.2022.1126639

**Published:** 2023-01-12

**Authors:** Linzhu Ren, Jieying Bai, Chang Li

**Affiliations:** ^1^ College of Animal Sciences, Key Lab for Zoonoses Research, Ministry of Education, Jilin University, Changchun, China; ^2^ Non-Human Primate Research Center, Institute of Molecular Medicine, Peking University, Beijing, China; ^3^ Research Unit of Key Technologies for Prevention and Control of Virus Zoonoses, Chinese Academy of Medical Sciences, Changchun Veterinary Research Institute, Chinese Academy of Agricultural Sciences, Changchun, China

**Keywords:** interferons, interferon stimulated genes, innate immunity, viral infection, interaction

Innate immunity, especially mediated by interferons (IFNs), is regarded as the first line of host immune protection against viral infection. As a strict intracellular parasite, the virus can enter the host cell through its receptor, thus surviving and proliferating in the cell (**Figure 1**). Since then, viruses and their hosts have been battling incessantly. Over evolutionary time, they have developed sophisticated and complicated regulation interactions (1, 2). The virus is constantly searching for the appropriate host and cells to replicate. Accordingly, the host has evolved strategies to eliminate these “enemies”. It uses an array of pattern recognition receptors (PRRs, such as TLRs) to detect unique pathogen-associated molecular patterns (PAMPs), followed by inducing IFNs, including type I interferons (IFNα and IFNβ) and type III interferons (IFNλ1–4). Like marshals, IFNs orchestrate and maneuver the production of hundreds of IFN-stimulated genes (ISGs). As reported, most ISGs exhibit antiviral activities, such as interferon-induced transmembrane proteins (IFITMs), Cholesterol 25-hydroxylase (CH25H), protein kinase R (PKR), etc. (1, 2). Therefore, the ISGs, like generals, are dispatched to the front line of the war to fight and inhibit viral infection in every step, from viral entry, replication, and assembly to egress. However, viruses also evolved multiple mechanisms to evade the inhibition of specific ISGs (**Figure 1**). To date, accumulating excellent publications have demonstrated the dynamic relationship between virus and host (3). However, many questions still need to be further uncovered.

**Figure 1:**
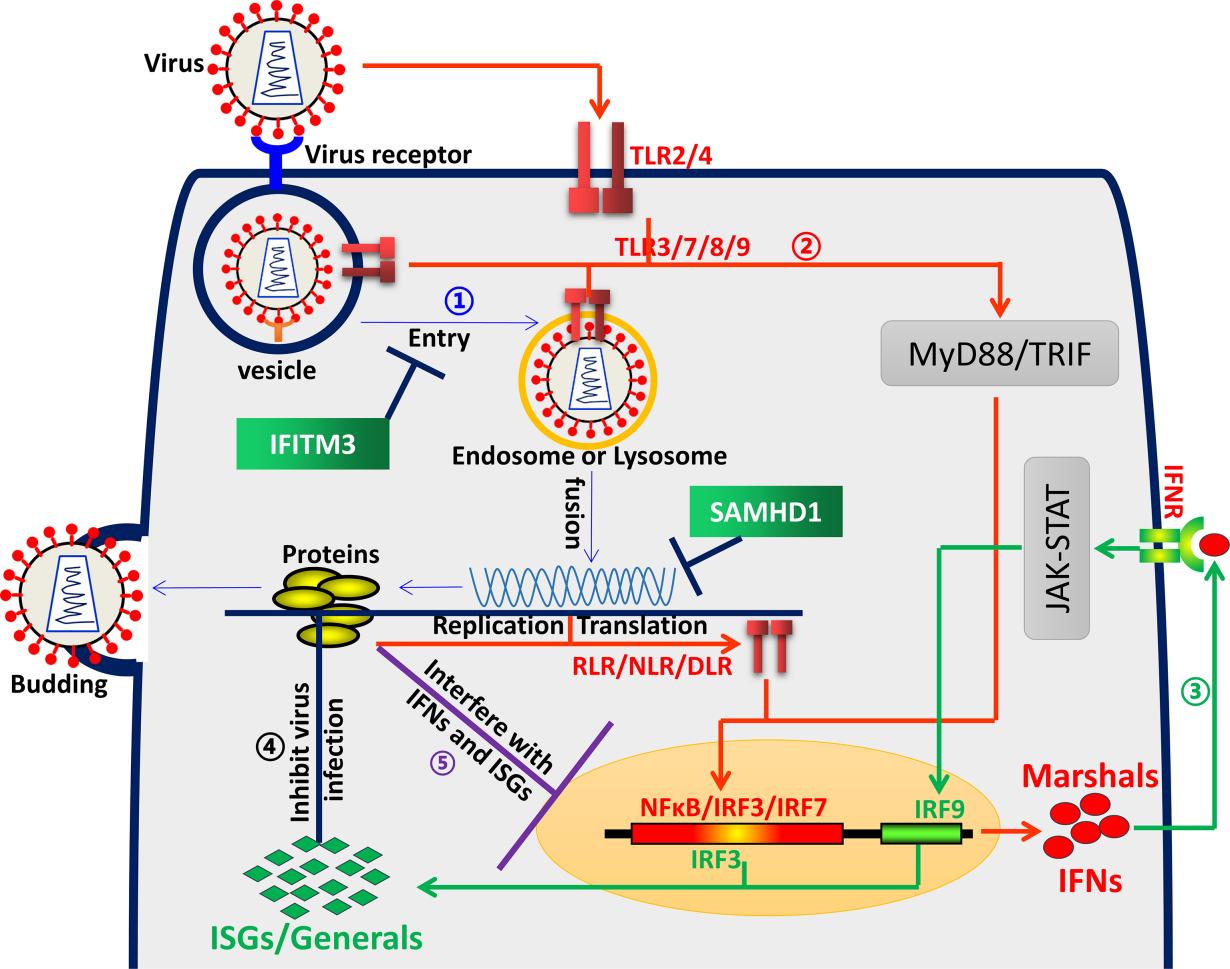
Virus and host interaction. ① Blue arrows represent the virus life cycle. A virus first binds to its receptor and entry cells depending on endocytic uptake, then delivers the viral genome into the cytosol and uses a repertoire of cellular processes to complete its replication, assembly and release. ② Red arrows represent the interferon induction process. The host uses an array of pattern recognition receptors (PRRs), such as TLRs, NLRs, etc., to detect unique pathogen-associated molecular patterns (PAMPs), followed by inducing expression of IFNs. ③ Green arrows represent the ISGs induction process. IFNs bind to cell surface receptors (IFNR), triggering a signal transduction cascade, producing hundreds of ISGs. ④ Black lines represent ISGs interfering with virus infection. ISGs delay or inhibit viral infection in different stages, such as entry, viral gene expression and genome amplification, viral particle assembly and egress, etc., with diverse mechanisms. ⑤ Purple lines represent virus antagonism towards ISGs. Viruses have evolved multiple evasion proteins, targeting IFN signal pathways through various sophisticated mechanisms and escaping IFN-ISG-mediated antiviral activities.

The goals of this collection are focused on studies of the complex interactions between ISGs and viruses. It is of great significance to elucidate the function and molecular mechanisms of action of known or new ISGs *in vitro* or *in vivo* to provide insights into viral pathogenesis, the scientific basis for novel vaccine design, and new targets for antiviral drug development.

To explore the expression of ISGs against viral infection, Song et al. performed differential transcriptomics. As a result, 90 shared upregulated ISGs were identified in porcine IPEC-J2 cells single or coinfected with porcine epidemic diarrhea virus (PEDV) and/or transmissible gastroenteritis virus (TGEV), among which 27 ISGs have antiviral activities. Furthermore, by analyzing many references and databases, they depicted the activity of ISGs in inhibiting or delaying virus proliferation at different stages of the virus cycle, indicating potential candidate targets for antiviral research. Moreover, swine IFITM3, one of the ISGs, can significantly inhibit the infection of PEDV, TGEV, and vesicular stomatitis virus (VSV) in different cells, indicating that it has broad-spectrum antiviral activity (Song et al.).

*In addition*, Ding et al. conducted a transcriptome analysis of primary macrophages induced by IFN-γ, a type II interferon that exhibits not only antibacterial but also antiviral activity in the Chinese sturgeon (*Acipenser sinensis*). A total of 4004 differential expression genes (DEGs) were enriched in the GO annotation, many of which were involved in immune-related processes. KEGG enrichment analysis showed that six signaling pathways are related to interferon regulation, emphasizing the importance of IFN-γ. Wang et al. evaluated the proteome in lung tissues of a cynomolgus monkey model in the early stage of severe acute respiratory syndrome coronavirus 2 (SARS-CoV-2) infection. They identified 44 proteins from all 194 significantly altered proteins, most encoded by ISGs. These results strongly indicate that IFN-ISG-mediated signaling pathways are essential in host resistance to viral infection. Besides, Dang et al. also established a high-through screening type I interferon-inducible antiviral effectors platform based on CRISPR/Cas9 knockout library with 1908 sgRNAs targeting 359 ISGs. Using VSV-eGFP as a model virus, a subset of the highest-ranking candidates, including known or novel host factors, were identified, demonstrating useful and promising methods to screen novel ISGs against a panel of porcine viruses.

As mentioned above, research on the functions and mechanisms of ISGs is crucial. Lang et al. provided a macroscopic overview of the characteristics, expressions, and mechanisms of ISGs in the central nervous system (CNS) and neurological diseases, including neurological inflammation, neuropsychiatric disorders, and neurodegenerative diseases, during viral infection. The authors also summarized up-to-date progress on viral infection in neurological disorders, exhibiting the broad promise application vision and high commercial value of ISGs as potential clinical biomarkers and therapeutic targets. Furthermore, IFITM3, a well-studied ISG, can inhibit a diverse range of pathogenic virus infections *in vivo* or *in vitro*. But the precise antiviral mechanisms remain unclear. Wen et al. reviewed the latest progress on the antiviral mechanism of IFITM3, the regulation mechanism by four post-translational modifications (PTMs), especially S-palmitoylation modification, providing helpful information for understanding IFITM3 antiviral mechanism and aiding in the design of therapeutics. An et al. reported that sterile alpha motif and histidine-aspartate domain-containing protein 1 (SAMHD1), another ISG, inhibited lipid bio-metabolic pathway to suppress flavivirus infection. Further studies showed that SAMHD1 could negatively regulate the expression of sterol regulatory element binding protein (SREBP1) and the formation of histidine aspartic acid-containing domain (HD), two critical factors associated with the lipid bio-metabolic pathway. These results revealed a novel mechanism of SAMHD1 and laid a new target for targeting lipid bio-metabolic pathway against the *Flaviviridae* family and other lipid-dependent viruses.

In summary, more and more antiviral ISGs have been discovered and studied with new technologies, such as unbiased screening approaches, differential transcriptome and proteome, affinity chromatography-mass spectrometry, proximity interactome, etc. The articles on this Research Topic provided new insights into the recent advances in ISGs. However, for most ISGs, the exact mechanisms of virus inhibition have not been clarified. Additionally, the vital importance of evaluating the effectiveness and safety of these ISGs *in vivo* models or human studies is highly summoned beyond mechanism. It may take more effort and time to get there, but it is hoped that many scientists will pay more attention to this field, solving and unveiling the most sophisticated antiviral defense systems to apply these ISGs in transgenic animal breeding or gene therapy as soon as possible.

## Author contributions

LR, JB, and CL wrote the draft and revised it critically for important intellectual content. All authors contributed to the article and approved the submitted version.
